# A Low-Cost RGB-D Sensing Front-End for Stable 3D Hand Landmark Reconstruction Using MediaPipe and ZED2 Stereo Depth

**DOI:** 10.3390/s26123730

**Published:** 2026-06-11

**Authors:** Laixin Peng, Tiansheng Liu, Bingwei He

**Affiliations:** 1 School of Mechanical Engineering and Automation, Fuzhou University, Fuzhou 350108, China; penglaixin@fzu.edu.cn (L.P.);; 2 Fujian Engineering Research Center of Joint Intelligent Medical Engineering, Fuzhou University, Fuzhou 350108, China

**Keywords:** RGB-D sensing, sensor fusion, 3D hand landmark reconstruction, stereo depth, MediaPipe, ZED2, hand motion tracking, temporal filtering, bone-length constraint

## Abstract

Stable three-dimensional hand landmark reconstruction using low-cost RGB-D sensors is important for human–computer interaction, robot teleoperation, and vision-based motion analysis. RGB-based hand landmark detectors provide stable semantic 2D landmarks, but their depth output is not a metric measurement in the physical camera coordinate system. Stereo cameras can provide metric depth, but direct landmark-level back-projection is sensitive to invalid pixels, local depth holes, boundary noise, and partial occlusion. To address these problems, this paper presents a lightweight RGB-D sensing front-end that combines MediaPipe semantic hand landmarks with ZED2 stereo depth. The proposed pipeline detects 21 semantic hand landmarks in the RGB image, obtains landmark-level metric depth from the aligned ZED2 depth map using local median sampling, reconstructs 3D landmarks by camera back-projection, and further applies exponential moving average filtering and a bone-length consistency constraint. Experiments were conducted on a self-collected SVO dataset containing 13 hand actions and 26 recorded sequences, and an additional checkerboard-based reference-distance validation was performed to evaluate the metric depth sampling and 3D back-projection component. Compared with single-pixel sampling, the 5×5 local median strategy slightly increased the valid-depth ratio from 0.9731 to 0.9738 and reduced the temporal smoothness metric from 1.7163 mm to 1.6902 mm. To further justify the temporal filtering choice, an additional comparison with the 1 Euro Filter was conducted using the reconstructed win5 trajectories. The 1 Euro Filter produced stronger smoothing, reducing the temporal smoothness metric to 0.196 mm, but also reduced the path-length ratio to 0.484, indicating substantial motion attenuation. EMA0.7 was therefore retained as a more balanced setting, reducing the temporal smoothness metric to 0.826 mm while maintaining a path-length ratio of 0.803. The BL0.5 bone-length constraint reduced the bone-length standard deviation from 2.0727 mm to 1.1995 mm with limited trajectory modification. The final configuration provides a practical low-cost RGB-D front-end for stable 3D hand landmark reconstruction under controlled indoor conditions.

## 1. Introduction

Three-dimensional hand landmark measurement is an important sensing component for human–computer interaction, robot teleoperation, augmented reality, and vision-based motion analysis [1,2,3]. Compared with full hand mesh reconstruction or marker-based motion capture systems, sparse hand landmarks provide a lightweight representation of hand motion and can be conveniently integrated into downstream interaction and robotic systems. However, obtaining stable and metric 3D hand landmarks with low-cost sensing devices remains challenging, especially when the hand undergoes fast motion, partial occlusion, or viewpoint changes.

Recent RGB-based hand landmark detectors provide an efficient solution for semantic hand localization from monocular images [1]. MediaPipe Hands is particularly attractive for practical applications because it can output 21 semantic hand landmarks with a fixed skeleton topology in real time [4,5,6]. These landmarks provide consistent anatomical correspondence across frames, including the wrist, finger joints, and fingertips. Nevertheless, the depth-related output of RGB-based models is usually a relative model prediction rather than a metric measurement in the physical camera coordinate system. Therefore, additional depth sensing is still required when real-scale 3D hand positions are needed. Recent validation studies on MediaPipe-based hand tracking also indicate that depth-enhanced frameworks can provide useful support for 3D hand movement measurement [7].

Depth and RGB-D sensing have been widely used for 3D hand pose estimation because depth measurements can reduce the scale ambiguity of monocular RGB input [2,8]. Existing studies have investigated depth-image-based regression, voxel representations, point-cloud representations, compact pose priors, RGB-D fusion networks, and stereo-camera-based hand pose estimation [9,10,11,12,13,14]. Large-scale hand pose datasets and benchmark systems, such as InterHand2.6M, have further promoted the development of learning-based 3D hand pose estimation methods with annotated 3D ground truth [3]. These methods demonstrate the importance of depth information, hand topology, and large-scale annotated data for recovering 3D hand structure.

However, many existing approaches are designed to predict complete hand pose or hand mesh parameters using trained regression models and benchmark datasets. They are not primarily designed as a transparent sensing front-end for combining semantic 2D landmarks with metric stereo depth measurements. In contrast, the objective of this work is to develop a lightweight and reproducible RGB-D sensing front-end that does not require training a new hand pose network. The proposed method focuses on landmark-level metric depth acquisition, temporal stability, and skeletal consistency under controlled indoor conditions rather than benchmark-level absolute 3D hand pose accuracy.

Stereo cameras provide a practical way to obtain metric depth in low-cost vision-based sensing systems [15,16]. By combining 2D hand landmarks with an aligned stereo depth map, each semantic landmark can be back-projected into the camera coordinate system. However, direct per-pixel depth sampling is sensitive to invalid pixels, local depth holes, boundary noise, foreground–background discontinuities, and small 2D localization errors, which are common challenges in depth-based hand pose estimation [2]. These problems are particularly evident near fingertips, thin finger regions, self-occluded areas, and fast hand motions. Therefore, directly reading the depth value at the landmark center pixel may lead to unstable 3D trajectories and inconsistent hand skeletons.

Besides depth acquisition, temporal continuity and skeletal consistency are also important for practical hand motion sensing. Temporal filtering is commonly used to reduce frame-to-frame jitter in interactive tracking signals, and the 1 Euro Filter is a representative lightweight method that adaptively balances jitter suppression and response delay [17]. In addition, structured hand representations, anatomical priors, and hand topology modeling have been used to improve the plausibility of reconstructed hand configurations [3,18,19]. Consumer-friendly RGB-D sensing has also been explored for markerless hand movement assessment, indicating the practical value of low-cost RGB-D systems for hand motion analysis [20]. Motivated by these observations, this study combines local median depth sampling, EMA-based temporal filtering, and a lightweight bone-length consistency constraint in a single RGB-D sensing pipeline.

This paper presents a low-cost RGB-D sensing front-end for stable 3D hand landmark reconstruction under controlled indoor conditions using MediaPipe semantic landmarks and ZED2 stereo depth. The proposed method first detects 2D semantic hand landmarks from the RGB image and then obtains landmark-level metric depth from the aligned ZED2 depth map. Instead of relying on a single depth pixel, local median depth sampling is used to reduce the influence of invalid or noisy depth values. The sampled depth values are then combined with camera intrinsic parameters to reconstruct 3D hand landmarks. EMA-based temporal filtering and a bone-length consistency constraint are further introduced to improve temporal continuity and skeletal stability.

The main contributions of this work are summarized as follows:

- A low-cost RGB-D sensing front-end is developed for metric 3D hand landmark reconstruction by combining the semantic 2D landmark topology of MediaPipe with the stereo depth measurements of the ZED2 camera.
- A landmark-level local median depth sampling strategy is evaluated to improve depth acquisition robustness under invalid depth pixels, local depth holes, and boundary fluctuations.
- A lightweight post-processing framework combining EMA-based temporal filtering and a bone-length consistency constraint is introduced to improve temporal continuity and skeletal stability without training a new hand pose regression network.
- A self-collected SVO dataset containing 13 hand actions and 26 recorded sequences is used to evaluate the effects of depth sampling, temporal filtering, bone-length correction, and lightweight filtering baselines through controlled ablation experiments and statistical analysis.

## 2. Materials and Methods

### 2.1. System Overview

The proposed method is designed as a lightweight RGB-D sensing front-end for stable RGB-D-based 3D hand landmark reconstruction under controlled indoor conditions. It combines the semantic 2D landmark topology provided by MediaPipe with the metric depth measurements obtained from the ZED2 stereo camera (Stereolabs, San Francisco, CA, USA). Given an RGB-D hand sequence, the pipeline outputs a sequence of 3D hand landmarks expressed in the left-camera coordinate system.

For each frame, the left RGB image is first processed by MediaPipe to detect 21 semantic 2D hand landmarks. The aligned ZED2 depth map is then used to obtain the metric depth value corresponding to each detected landmark. Instead of directly using the depth value at a single pixel, a local median sampling strategy is applied around each landmark to reduce the influence of invalid depth pixels, local depth holes, and boundary noise. The sampled depth and the camera intrinsic parameters are then used to back-project the 2D landmarks into 3D space.

After geometric reconstruction, two lightweight regularization modules are applied. EMA-based temporal filtering is used to suppress frame-to-frame fluctuations in the reconstructed trajectories, and a bone-length consistency constraint is used to reduce temporal variations in the reconstructed hand skeleton. Therefore, the complete pipeline consists of six steps: semantic landmark detection, stereo depth acquisition, local depth sampling, 3D back-projection, temporal filtering, and bone-length consistency correction.

### 2.2. Hardware Platform and SVO Data Acquisition

The experimental platform consisted of a ZED2 stereo camera and a computer workstation. Hand motion sequences were recorded in the SVO format with stereo acquisition enabled. The left camera view was used as the primary RGB input for MediaPipe-based landmark detection. According to the session metadata, the image resolution was 1280×720, and the nominal frame rate was 30 fps. During data acquisition, the hand was placed approximately 30 cm from the camera. The camera was fixed in a front-facing stereo view, and all sequences were recorded under stable indoor LED illumination with a plain dark desk background. The right hand was used in the recorded sequences, and table support was allowed during recording. The camera configuration kept auto-exposure and auto-focus enabled.

The processing program was implemented in Python using OpenCV, MediaPipe, and the ZED Python API. The software environment included Python 3.14.3, OpenCV 4.13.0, MediaPipe 0.10.33, and ZED SDK 5.2.3. The experiments were conducted on a workstation equipped with an AMD Ryzen 7 5800H CPU with 8 cores and 16 logical processors, an NVIDIA GeForce RTX 3060 Laptop GPU, and 16 GB RAM. The implementation was not specifically optimized for GPU acceleration.

Offline SVO playback was used for all subsequent experiments. This design ensured that different depth sampling, temporal filtering, and bone-length constraint settings were evaluated using the same RGB images, depth maps, and temporal sequences. Therefore, the observed differences among the compared strategies were mainly caused by the processing modules rather than by variations in data acquisition.

### 2.3. MediaPipe-Based Semantic Landmark Detection

MediaPipe Hand Landmarker is used to detect semantic hand landmarks from the left RGB image [4,5,6]. For each detected hand, MediaPipe provides 21 landmarks with fixed semantic definitions, including the wrist, finger joints, and fingertips. These landmarks provide a consistent hand skeleton topology and make it possible to associate each image point with a specific anatomical location.

Let the normalized image coordinate of the *i*-th landmark be denoted as

(1) $$p_{i}^{n}=\left(x_{i}^{n},y_{i}^{n}\right),$$

where $x_{i}^{n}$ and $y_{i}^{n}$ are normalized by the image width and height, respectively. Given an image with width *W* and height *H*, the corresponding pixel coordinate is computed as

(2) $$u_{i}=x_{i}^{n}W,$$

(3) $$v_{i}=y_{i}^{n}H.$$

The pixel coordinate of the *i*-th landmark is therefore represented as

(4) $$p_{i}=\left(u_{i},v_{i}\right).$$

This conversion follows the image-coordinate convention used by MediaPipe landmark outputs, in which normalized landmark coordinates are mapped to pixel coordinates according to the image width and height [4,6].

It should be noted that the depth-related output of MediaPipe is a relative model prediction rather than a metric depth measurement in the physical camera coordinate system. Therefore, this study does not use the MediaPipe depth output as the final 3D depth. MediaPipe is used only to provide semantic 2D landmark locations, while the metric depth of each landmark is obtained from the aligned ZED2 stereo depth map.

### 2.4. ZED2 Stereo Depth Acquisition

The ZED2 stereo camera is used to provide metric depth information for the detected 2D hand landmarks [15,16]. For each SVO sequence, the left RGB image and the corresponding depth map are read frame by frame. The depth map is aligned with the left camera image, so that each pixel location in the RGB image has a corresponding depth value when the stereo matching result is valid. This formulation follows the standard aligned RGB-D sensing setting, where a metric depth value is associated with the corresponding pixel in the reference camera image [16,21].

Let the depth map at frame *t* be denoted as

(5) $$D^{t}\left(u,v\right),$$

where $D^{t}\left(u,v\right)$ represents the metric depth value at pixel location (u,v). In this study, the depth value is treated as the *Z* coordinate in the left-camera coordinate system and is used for 3D back-projection together with the camera intrinsic parameters.

Stereo depth maps may contain invalid values, holes, and local fluctuations, especially near object boundaries, thin finger regions, self-occluded areas, and foreground–background transitions. Directly using the depth value at a single landmark pixel may therefore lead to unstable 3D landmark estimates. To address this problem, local depth sampling strategies are introduced to obtain a more robust landmark-level depth value from the neighborhood of each 2D landmark.

### 2.5. Local Median Depth Sampling

After obtaining the 2D semantic landmarks and the aligned ZED2 depth map, a metric depth value must be assigned to each landmark before 3D back-projection. A straightforward strategy is to directly read the depth value at the landmark pixel. However, stereo depth maps often contain invalid pixels, local holes, boundary noise, and foreground–background discontinuities. When a landmark is located near a fingertip boundary, a thin finger region, or a partially occluded area, single-pixel depth sampling may produce unstable or invalid depth measurements.

**Figure 1:**
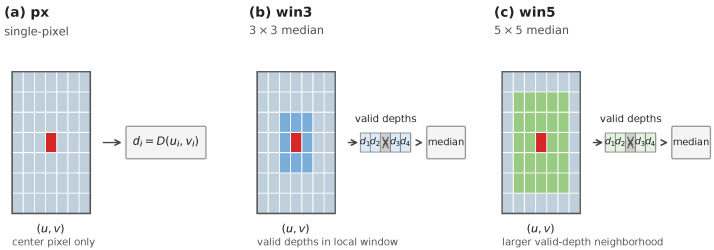
Illustration of local depth sampling strategies for hand landmarks. (**a**) The px strategy uses only the depth value at the landmark center pixel. (**b**) The win3 strategy uses the median of valid depth values within a 3×3 neighborhood. (**c**) The win5 strategy uses the median of valid depth values within a 5×5 neighborhood.

To improve landmark-level depth acquisition, this study evaluates three local depth sampling strategies: single-pixel sampling, 3×3 median sampling, and 5×5 median sampling. The three strategies differ only in the way the landmark depth value $d_{i}$ is obtained, while the MediaPipe landmark detection results, camera intrinsic parameters, and 3D back-projection model remain unchanged. Figure 1 illustrates the three evaluated sampling strategies.

Let the pixel coordinate of the *i*-th landmark be denoted as

(6) $$p_{i}=\left(u_{i},v_{i}\right),$$

and let the depth map be denoted as

(7) D(u,v),

where D(u,v) represents the metric depth value at pixel location (u,v).

In the single-pixel strategy, denoted as px, the landmark depth is directly obtained from the depth map:

(8) $$d_{i}=D\left(u_{i},v_{i}\right).$$

If this depth value is non-finite, missing, or less than or equal to zero, the corresponding landmark is marked as invalid in the current frame.

For local median sampling, a square window is defined around the landmark pixel. For a window radius *r*, the local neighborhood is defined as

(9) $$\Omega_{i}^{r}=\left\{\left(u,v\right)|u\in \left[u_{i}-r,u_{i}+r\right],\;v\in \left[v_{i}-r,v_{i}+r\right]\right\}.$$

The valid depth set within this neighborhood is defined as

(10) $$D_{i}^{r}=\left\{D\left(u,v\right)|\left(u,v\right)\in \Omega_{i}^{r},\;D\left(u,v\right)>0\right\}.$$

If $D_{i}^{r}$ is non-empty, the landmark depth is computed as the median of the valid depth values. Median filtering is commonly used to reduce the influence of isolated outliers and impulse-like local noise in image and depth measurements [22]:

(11) $$d_{i}=\mathrm{median}\left(D_{i}^{r}\right).$$

If no valid depth value exists within the local window, the landmark is marked as invalid in the current frame.

In this study, r=1 corresponds to the 3×3 strategy, denoted as win3, and r=2 corresponds to the 5×5 strategy, denoted as win5. Compared with px, local median sampling can reduce the influence of isolated invalid or noisy depth pixels. However, a larger window may also include background depth values near hand boundaries or self-occluded regions. Therefore, px, win3, and win5 are quantitatively compared in the experiments to determine a suitable depth sampling strategy for the subsequent 3D hand landmark measurement pipeline.

### 2.6. 3D Back-Projection

After obtaining the depth value $d_{i}$ for the *i*-th landmark, the corresponding 2D pixel coordinate is back-projected into the camera coordinate system using the intrinsic parameters of the ZED2 left camera. This back-projection follows the standard pinhole camera model used in projective geometry and RGB-D reconstruction [21,23]. Let $f_{x}$ and $f_{y}$ denote the focal lengths, and let $c_{x}$ and $c_{y}$ denote the principal point coordinates. The 3D coordinate of the *i*-th landmark is computed as

(12) $$X_{i}=\frac{\left(u_{i}-c_{x}\right)d_{i}}{f_{x}},$$

(13) $$Y_{i}=\frac{\left(v_{i}-c_{y}\right)d_{i}}{f_{y}},$$

(14) $$Z_{i}=d_{i}.$$

Thus, the 3D hand landmark in the camera coordinate system is represented as

(15) $$P_{i}=\left(X_{i},Y_{i},Z_{i}\right).$$

It should be noted that the three sampling strategies only affect the estimation of the depth value $d_{i}$. They do not change the MediaPipe-based 2D landmark detection results or the camera back-projection model. This design ensures that the subsequent comparison focuses only on the influence of local depth sampling window size.

All reconstructed 3D landmarks are expressed in the left-camera coordinate system. The depth value provided by the ZED2 camera is treated as the camera-frame *Z* coordinate, and all reported 3D coordinates and derived metrics are converted to millimeters for quantitative analysis.

### 2.7. EMA-Based Temporal Filtering

After 3D back-projection, the reconstructed landmark trajectories may still contain frame-to-frame fluctuations caused by stereo depth noise, small 2D landmark localization errors, and short-term tracking instability. Temporal filtering is commonly used to reduce such fluctuations in interactive tracking signals [17]. The EMA filter is a first-order recursive smoothing filter that updates the filtered signal by combining the current observation with the previous filtered estimate [24]. In this study, an exponential moving average (EMA) filter is applied to the reconstructed 3D landmark sequence as a lightweight temporal smoothing module.

Let $P_{i}^{t}$ denote the reconstructed 3D position of the *i*-th landmark at frame *t*, and let ${\hat{P}}_{i}^{t}$ denote the filtered position. The EMA filter is defined as

(16) $${\hat{P}}_{i}^{t}=\alpha P_{i}^{t}+\left(1-\alpha \right){\hat{P}}_{i}^{t-1},$$

where α∈(0,1] controls the filtering strength. A smaller α produces stronger smoothing but may introduce larger temporal lag and motion attenuation, whereas a larger α preserves rapid hand motion better but provides weaker jitter suppression.

The filter is applied independently to the three coordinate components of each valid 3D landmark. When a landmark is invalid in the current frame, it is not used to update the corresponding filtered trajectory. In the experiments, three values of α, namely 0.3, 0.5, and 0.7, are evaluated to analyze the trade-off between temporal smoothness and motion preservation. The unfiltered win5 output is used as the baseline for comparison.

### 2.8. Additional Temporal Filtering Baseline

To further evaluate the selected EMA-based temporal filtering strategy, the 1 Euro Filter was implemented as an additional lightweight temporal filtering baseline [17]. The 1 Euro Filter is commonly used in interactive tracking because it adaptively adjusts the cutoff frequency according to the estimated signal velocity, thereby addressing the trade-off between jitter suppression and motion responsiveness.

In this study, the 1 Euro Filter was applied independently to the three coordinate components of each valid reconstructed 3D landmark, using the same win5 trajectories as the input. The filtering parameters were set to mincutoff=1.0, β=0.01, and dcutoff=1.0. The filtered output was compared with the unfiltered win5 trajectory and the EMA0.7 output using the same sequence-level metrics, including *Z*-axis standard deviation, temporal smoothness, bone-length standard deviation, path-length ratio, and computational overhead.

### 2.9. Bone-Length Consistency Constraint

Although temporal filtering can reduce frame-to-frame fluctuations, each 3D landmark is still reconstructed independently from the sampled depth value. As a result, the temporal length of a reconstructed hand bone may vary across frames, even though the anatomical bone length of the same subject should remain approximately constant within a short sequence. Structured hand representations, anatomical priors, and hand topology modeling are commonly used to improve the plausibility of 3D hand pose estimation [3,18,19]. In this study, a lightweight bone-length consistency constraint is applied after EMA filtering to reduce temporal skeletal variation.

The hand skeleton is represented by a set of connected bones B following the MediaPipe hand topology. The bone set follows the semantic hand topology used by MediaPipe and is consistent with common kinematic-chain representations of hand landmarks [4,6,18]. Specifically, the bone set is defined as

(17) $$\begin{array}{cc} B=\{ & \;(0,1),(1,2),(2,3),(3,4),\\ \; & \;(0,5),(5,6),(6,7),(7,8),\\ \; & \;(0,9),(9,10),(10,11),(11,12),\\ \; & \;(0,13),(13,14),(14,15),(15,16),\\ \; & \;(0,17),(17,18),(18,19),(19,20)\}. \end{array}$$

For a bone connecting the parent landmark *i* and the child landmark *j*, the bone length at frame *t* is computed as

(18) $$l_{ij}^{t}={∥{\hat{P}}_{j}^{t}-{\hat{P}}_{i}^{t}∥}_{2},$$

where ${\hat{P}}_{i}^{t}$ and ${\hat{P}}_{j}^{t}$ denote the EMA-filtered 3D positions of the parent and child landmarks, respectively. The reference length of each bone is estimated as the median bone length over valid frames:

(19) $${\bar{l}}_{ij}={\mathrm{median}}_{t}\left(l_{ij}^{t}\right).$$

The median is used to reduce the influence of occasional outliers caused by invalid depth values, local depth noise, or temporary tracking instability.

Given the reference length ${\bar{l}}_{ij}$, the current bone vector is defined as

(20) $$v_{ij}^{t}={\hat{P}}_{j}^{t}-{\hat{P}}_{i}^{t}.$$

The fully projected child landmark position is computed by preserving the current bone direction while enforcing the reference bone length:

(21) $$P_{j,\mathrm{proj}}^{t}={\hat{P}}_{i}^{t}+{\bar{l}}_{ij}\frac{v_{ij}^{t}}{{∥v_{ij}^{t}∥}_{2}}.$$

To avoid over-constraining the reconstructed motion, a correction strength λ is introduced:

(22) $$P_{j,\mathrm{corr}}^{t}=\left(1-\lambda \right){\hat{P}}_{j}^{t}+\lambda P_{j,\mathrm{proj}}^{t}.$$

When λ=0, no bone-length correction is applied. When λ=1, the child landmark is fully projected onto the reference bone length. In the experiments, two correction strengths are evaluated: λ=0.5 and λ=1.0, denoted as BL0.5 and BL1, respectively. BL0.5 applies a moderate correction toward the reference bone length, whereas BL1 fully enforces the reference length. These two settings are compared to analyze the trade-off between skeletal consistency and motion preservation.

### 2.10. Dataset and Evaluation Metrics

#### 2.10.1. SVO Dataset Collection and Scenario Design

A self-collected SVO dataset was built to evaluate the proposed RGB-D hand landmark measurement pipeline under different hand poses and motion conditions. During recording, the camera position, background, illumination, and hand motion region were kept consistent. All sequences were recorded using the right hand of the same subject. Each sequence started and ended with a short stable posture to reduce the influence of transient motion at the sequence boundaries.

The dataset contains 13 hand actions, and each action was recorded twice as t1 and t2, resulting in 26 SVO sequences. The actions were designed to cover four types of scenarios: static postures, dynamic motions, self-occlusion cases, and recovery cases. The static sequences were used to evaluate basic depth stability under limited motion, while the dynamic, occlusion, and recovery sequences were used to examine the behavior of the proposed pipeline under more challenging conditions. Table 1 summarizes the recorded actions and their purposes.

For controlled ablation, each SVO sequence was processed using the compared configurations while keeping the input RGB images, depth maps, and temporal order unchanged. The depth sampling experiment compared px, win3, and win5. The temporal filtering experiment was then performed on the selected depth sampling output using EMA parameters of 0.3, 0.5, and 0.7. Finally, the bone-length consistency experiment evaluated two correction strengths, BL0.5 and BL1. This experimental design allowed the contribution of each module to be analyzed separately.

It should be noted that the current dataset is used for preliminary system validation rather than for large-scale benchmark evaluation. Since no external motion-capture ground truth was used, the evaluation focuses on detection availability, depth availability, temporal stability, skeletal consistency, and motion preservation.

#### 2.10.2. Evaluation Metrics

Several metrics were used to evaluate the depth sampling strategies and the subsequent optimization modules. Detection rate and valid-depth ratio measure the availability of hand landmark detection and stereo depth acquisition. The *Z*-axis standard deviation, temporal smoothness, and bone-length standard deviation measure the stability of the reconstructed 3D landmark sequence. The path-length ratio measures motion preservation after filtering or correction, and the correction displacement quantifies the magnitude of the bone-length correction. All depth-related and 3D geometric metrics are reported in millimeters.

Let *T* denote the number of frames in a sequence and $N_{l}=21$ denote the number of MediaPipe hand landmarks. The detection rate is defined as

(23) $$R_{\det}=\frac{1}{T}\sum_{t=1}^{T}I_{\det}^{t},$$

where $I_{\det}^{t}=1$ if the hand is detected at frame *t*, and $I_{\det}^{t}=0$ otherwise.

For detected frames, the valid-depth ratio is defined as

(24) $$R_{\mathrm{valid}}=\frac{1}{T_{d}N_{l}}\sum_{t=1}^{T_{d}}\sum_{i=1}^{N_{l}}I_{\mathrm{valid}}^{t,i},$$

where $T_{d}$ is the number of detected frames and $I_{\mathrm{valid}}^{t,i}=1$ if the *i*-th landmark has a valid depth value at frame *t*.

The *Z*-axis standard deviation is used to evaluate depth-direction jitter:

(25) $$\sigma_{Z}=\frac{1}{N_{l}}\sum_{i=1}^{N_{l}}{\mathrm{std}}_{t}\left(Z_{i}^{t}\right),$$

where $Z_{i}^{t}$ is the depth coordinate of the *i*-th landmark at frame *t*.

Temporal smoothness is computed using the second-order difference of the 3D landmark trajectory:

(26) $$S_{\mathrm{temp}}=\frac{1}{N_{l}\left(T_{d}-2\right)}\sum_{i=1}^{N_{l}}\sum_{t=2}^{T_{d}-1}{∥P_{i}^{t+1}-2P_{i}^{t}+P_{i}^{t-1}∥}_{2}.$$

A lower value indicates a smoother local trajectory.

For a bone connecting landmarks *i* and *j*, the bone length at frame *t* is defined as

(27) $$l_{ij}^{t}={∥P_{j}^{t}-P_{i}^{t}∥}_{2}.$$

The bone-length standard deviation is then computed as

(28) $$\sigma_{\mathrm{bone}}=\frac{1}{\left|B\right|}\sum_{(i,j)\in B}{\mathrm{std}}_{t}\left(l_{ij}^{t}\right),$$

where B denotes the bone set defined by the MediaPipe hand topology. A lower value indicates better temporal skeletal consistency.

To evaluate whether temporal filtering or structural correction excessively changes the original motion, the path-length ratio is computed as

(29) $$R_{\mathrm{path}}=\frac{\sum_{i=1}^{N_{l}}\sum_{t=2}^{T_{d}}{∥P_{i,\mathrm{out}}^{t}-P_{i,\mathrm{out}}^{t-1}∥}_{2}}{\sum_{i=1}^{N_{l}}\sum_{t=2}^{T_{d}}{∥P_{i,\mathrm{base}}^{t}-P_{i,\mathrm{base}}^{t-1}∥}_{2}},$$

where $P_{i,\mathrm{base}}^{t}$ and $P_{i,\mathrm{out}}^{t}$ denote the baseline and processed trajectories, respectively. A value closer to 1 indicates better preservation of the original trajectory length.

For bone-length correction, the correction displacement is defined as

(30) $$D_{\mathrm{corr}}=\frac{1}{N_{l}T_{d}}\sum_{i=1}^{N_{l}}\sum_{t=1}^{T_{d}}{∥P_{i,\mathrm{corr}}^{t}-P_{i,\mathrm{in}}^{t}∥}_{2},$$

where $P_{i,\mathrm{in}}^{t}$ and $P_{i,\mathrm{corr}}^{t}$ are the landmark positions before and after bone-length correction. A lower value indicates a smaller correction magnitude.

Table 2 summarizes the metrics used in the experiments and their preferred directions.

### 2.11. Statistical Analysis

Because the number of evaluated sequences was limited and normality could not be assumed, paired Wilcoxon signed-rank tests were used to compare sequence-level metrics between paired methods. For the depth sampling experiment, paired tests were performed between the single-pixel baseline and the 5×5 local median sampling strategy using valid-depth ratio, *Z*-axis standard deviation, temporal smoothness, and bone-length standard deviation. For the temporal filtering comparison, paired tests were performed among the unfiltered win5 baseline, EMA0.7, and the 1 Euro Filter. For each statistical test, only sequences with valid paired metric values were included. Therefore, the effective sample size *N* is reported for each comparison.

### 2.12. Runtime Evaluation

Runtime was evaluated during offline SVO playback on the same workstation used for the experiments. The processing time per frame and the corresponding processing FPS were recorded for the win5 reconstruction stage. In addition, the computational overhead introduced by EMA0.7 and the 1 Euro Filter was measured separately. The runtime analysis was used to evaluate the computational cost of the proposed lightweight post-processing modules. It should be noted that the implementation was evaluated in offline SVO playback mode and was not specifically optimized for real-time deployment.

### 2.13. Calibrated Reference-Distance Validation

To provide an external metric reference for the RGB-D reconstruction component, a calibrated checkerboard was used for reference-distance validation. The checkerboard contained 12×9 squares, corresponding to 11×8 inner corners, and the measured square size was 15.0 mm. The board was fixed on a planar support and recorded by the ZED2 camera at five camera-to-board settings, including frontal and tilted viewpoints.

For each recorded SVO sequence, checkerboard inner corners were detected in the left camera image using OpenCV. The aligned ZED2 depth map was then used to obtain the metric depth value at each detected corner. To remain consistent with the selected win5 setting in the hand landmark experiments, a 5×5 local median depth sampling strategy was applied around each detected checkerboard corner. Each corner was then back-projected into the left-camera coordinate system using the ZED2 intrinsic parameters.

Pairwise 3D distances between selected reconstructed checkerboard corners were compared with their known physical distances computed from the measured square size. The mean absolute error (MAE), root mean square error (RMSE), mean signed error, error standard deviation, median absolute error, and maximum absolute error were calculated. This validation experiment was designed to evaluate the metric depth sampling and 3D back-projection component using calibrated reference distances, rather than to claim full articulated hand-pose ground-truth accuracy.

## 3. Results

### 3.1. Comparison of Depth Sampling Strategies

The first experiment compares three landmark-level depth sampling strategies: single-pixel sampling (px), 3×3 local median sampling (win3), and 5×5 local median sampling (win5). The purpose of this experiment is to evaluate how the local depth sampling scale affects depth availability, depth-direction stability, temporal continuity, and skeletal consistency. All three strategies use the same MediaPipe detection results, ZED2 depth maps, and back-projection model; therefore, the differences in the results are caused only by the depth sampling strategy.

Table 3 presents the overall quantitative results. The detection rates are identical because the sampling strategy is applied after MediaPipe hand detection and does not affect the detection stage. Compared with px, both win3 and win5 slightly increase the valid-depth ratio and reduce the temporal smoothness metric. Among the three strategies, win5 obtains the highest average valid-depth ratio and the lowest average temporal smoothness value in the overall comparison. The differences in *Z*-axis standard deviation and bone-length standard deviation are relatively small, indicating that local median sampling mainly affects landmark-level depth availability and short-term temporal continuity.

**Figure 2:**
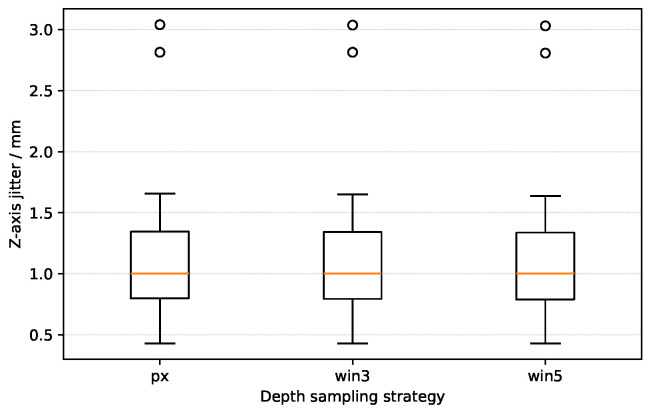
Comparison of *Z*-axis jitter under different depth sampling strategies in static scenarios. Outliers indicate frames or landmark trajectories with larger depth-direction deviations, which may be caused by local stereo depth holes, boundary fluctuations, or temporary landmark localization instability even under nominally static hand postures.

Figure 2 compares the *Z*-axis jitter under static scenarios. The three strategies show similar distributions, suggesting that single-pixel sampling can already provide relatively stable depth measurements when the hand remains still and occlusion is limited. Therefore, static scenarios mainly verify the basic stability of the depth acquisition process, while more challenging scenarios are needed to further compare the sampling strategies.

The outliers in Figure 2 mainly reflect occasional large depth-direction deviations in otherwise static sequences. Such deviations are likely caused by local stereo depth holes, boundary fluctuations near fingertips or finger edges, and small MediaPipe landmark localization variations. Therefore, these outliers indicate local instability in landmark-level RGB-D reconstruction rather than global hand motion. This observation further supports the use of local median depth sampling to reduce the influence of isolated invalid or unstable depth pixels.

**Figure 3:**
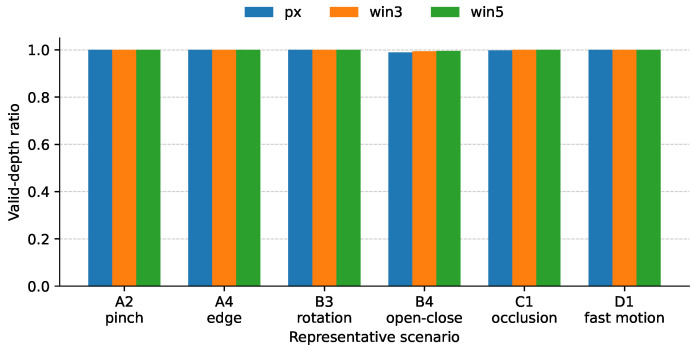
Comparison of valid-depth ratio under different depth sampling strategies in representative challenging scenarios. The figure was reformatted with a non-overlapping legend to improve visual readability.

To further evaluate the sampling strategies under more difficult conditions, six representative scenarios were selected, including fine pinching, boundary-view placement, wrist rotation, finger opening and closing, self-occlusion, and rapid motion. Figure 3 shows the valid-depth ratio in these scenarios. In several cases, win3 and win5 achieve higher or more stable valid-depth ratios than px, indicating that local-window sampling can compensate for invalid depth values at individual landmark pixels. However, the improvement in valid-depth ratio is relatively small in magnitude, and it should be interpreted together with temporal smoothness and statistical testing rather than as a standalone indicator of reconstruction accuracy.

**Figure 4:**
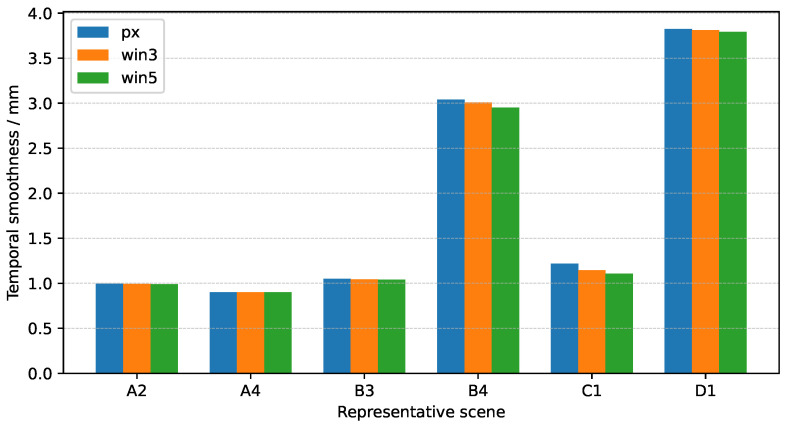
Comparison of temporal smoothness under different depth sampling strategies in representative challenging scenarios.

Figure 4 reports the temporal smoothness results in the selected challenging scenarios. win5 yields lower smoothness values in most selected cases, indicating better temporal continuity among the three strategies under challenging hand motion and occlusion conditions.

**Figure 5:**
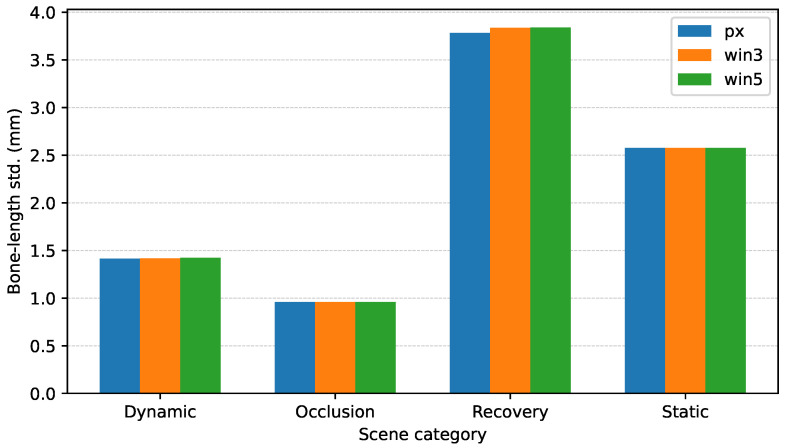
Comparison of bone-length standard deviation under different depth sampling strategies.

Figure 5 compares the bone-length stability under different depth sampling strategies. The differences among the three strategies are relatively limited, suggesting that local depth sampling alone does not explicitly enforce skeletal structural consistency. Nevertheless, the result provides a useful reference for evaluating whether the depth sampling strategy affects the temporal stability of the reconstructed hand skeleton.

Table 4 summarizes the number of best-performing sequences under different evaluation metrics. Tied best values are counted for all corresponding methods. win5 obtains the largest number of best-performing sequences in valid-depth ratio and temporal smoothness, and it also shows more favorable results in bone-length standard deviation than the other two strategies. In contrast, px performs better in *Z*-axis standard deviation for some sequences, suggesting that single-pixel sampling can still be stable in certain static or locally stable cases.

Overall, win5 provides the most favorable average performance in depth availability and temporal continuity among the three sampling strategies. Although local depth sampling alone does not explicitly constrain the hand skeleton, win5 also shows relatively favorable bone-length stability in the sequence-level comparison. Therefore, win5 is used as the depth sampling configuration for the subsequent temporal filtering and bone-length constraint experiments.

To further clarify whether the observed 5×5 sampling improvement was statistically supported, paired Wilcoxon signed-rank tests were performed between the single-pixel sampling baseline px and the win5 strategy using sequence-level metrics. The tests were performed on 11 valid paired segments with available metrics. Table 5 summarizes the results. The temporal smoothness metric was significantly reduced by win5 compared with px (p=0.001953), indicating improved temporal continuity. However, the differences in valid-depth ratio, *Z*-axis standard deviation, and bone-length standard deviation were not statistically significant. Therefore, the 5×5 sampling strategy is interpreted as providing a modest but statistically supported improvement in temporal continuity, rather than a large overall accuracy gain.

### 3.2. Calibrated Reference-Distance Validation Results

To provide an external metric reference for the RGB-D reconstruction component, calibrated checkerboard-based reference-distance validation was performed. Five SVO sequences were recorded at different camera-to-board distances and viewpoints. The checkerboard was successfully detected in all processed frames, and the mean valid corner ratio was 1.000.

Table 6 summarizes the validation results. Across 542,276 valid pairwise distance measurements, the proposed win5-based depth sampling and 3D back-projection produced an overall MAE of 0.181 mm and an RMSE of 0.225 mm. The mean signed error was −0.150 mm, indicating a slight underestimation of the reconstructed reference distances. The maximum absolute error was 1.099 mm. These results indicate that the metric depth sampling and camera back-projection component can recover calibrated reference distances with sub-millimeter average error under the tested indoor conditions.

**Figure 6:**
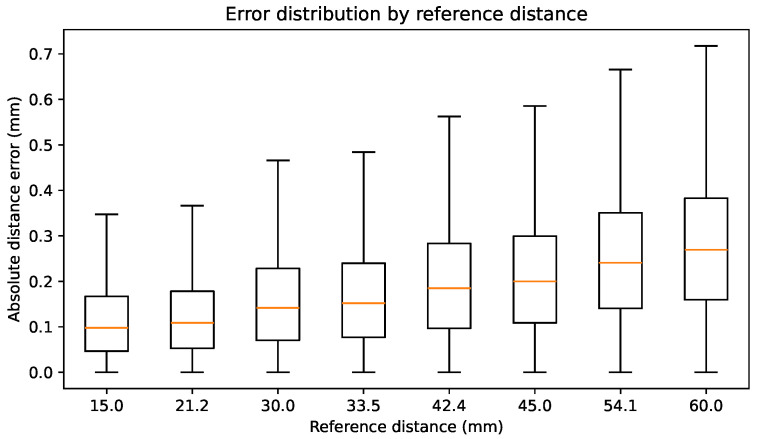
Distribution of absolute distance errors under different calibrated reference distances. The error increases slightly with the reference distance but remains below 0.3 mm in terms of MAE for all evaluated reference-distance groups.

Figure 6 shows the distribution of absolute distance errors under different calibrated reference distances.

The error increased slightly with the reference distance. The MAE increased from 0.117 mm at 15 mm to 0.279 mm at 60 mm, which is consistent with the accumulation of metric reconstruction error over longer pairwise distances. This experiment provides an external calibrated reference for the metric RGB-D reconstruction component. However, because the checkerboard is a rigid planar target, this validation does not replace motion-capture-level ground truth for articulated hand landmarks.

### 3.3. Evaluation of EMA Temporal Filtering

The second experiment evaluates the effect of EMA-based temporal filtering on the 3D hand landmark trajectories. Based on the previous depth sampling experiment, win5 is used as the input configuration. Three EMA parameters, 0.3, 0.5, and 0.7, are compared with the unfiltered win5 baseline. The purpose of this experiment is to analyze the trade-off between trajectory smoothing and motion preservation.

Table 7 presents the overall quantitative results. Compared with the unfiltered win5 baseline, all EMA settings reduce the temporal smoothness metric, indicating that EMA filtering can suppress frame-to-frame fluctuations in the reconstructed 3D trajectories. Among the three filtered results, EMA 0.3 obtains the lowest temporal smoothness value, decreasing the metric from 1.6902 mm to 0.4950 mm. However, its path-length ratio is only 0.5622, indicating a clear reduction in the reconstructed trajectory length.

EMA 0.7 provides a weaker smoothing effect than EMA 0.3 and EMA 0.5, but it better preserves the original motion trajectory. Specifically, EMA 0.7 reduces the temporal smoothness metric from 1.6902 mm to 1.0995 mm while maintaining a path-length ratio of 0.7999. This result suggests that EMA 0.7 provides a more balanced setting between jitter suppression and motion preservation.

**Figure 7:**
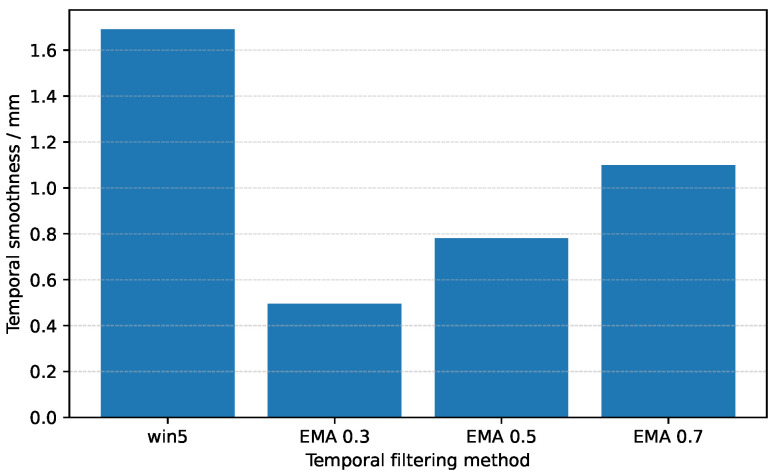
Temporal smoothness comparison of different filtering settings.

Figure 7 compares the overall temporal smoothness values. The smoothness metric decreases as the EMA parameter becomes smaller, confirming that a smaller α produces stronger temporal smoothing. However, this improvement in smoothness should be interpreted together with the path-length ratio, because stronger smoothing may also attenuate valid hand motion.

**Figure 8:**
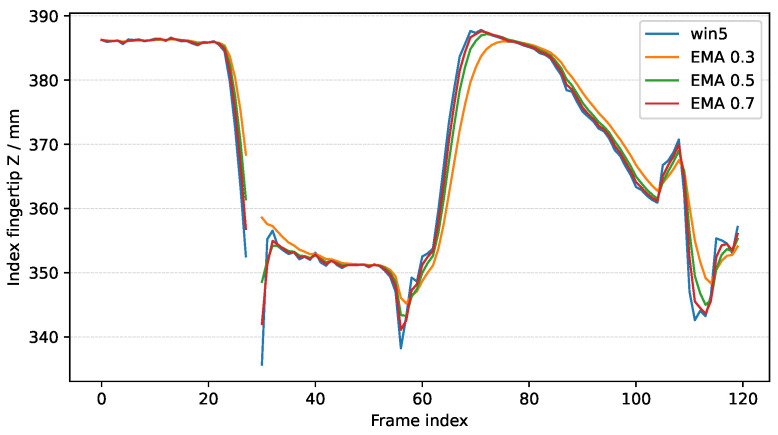
Index fingertip *Z*-axis trajectory under different EMA settings in the B4 sequence.

A representative *Z*-axis trajectory of the index fingertip from the B4 sequence is shown in Figure 8. The unfiltered win5 trajectory contains local fluctuations. EMA 0.3 produces the smoothest curve, but it also shows visible lag and amplitude attenuation during rapid depth changes. EMA 0.7 follows the original trajectory more closely while still reducing high-frequency fluctuations. This observation is consistent with the path-length ratio results in Table 7.

Table 8 reports the number of best-performing sequences under different temporal filtering metrics. EMA 0.3 obtains the best temporal smoothness in all evaluated sequences, while the unfiltered win5 baseline best preserves the trajectory length. EMA 0.7 does not achieve the strongest smoothing, but it provides better motion preservation than EMA 0.3 and EMA 0.5.

Overall, EMA 0.3 provides the strongest jitter suppression but also causes substantial motion attenuation. EMA 0.7 provides a more practical trade-off between temporal smoothness and motion preservation. Therefore, EMA 0.7 is used in the subsequent bone-length consistency experiment.

### 3.4. Comparison with the 1 Euro Filter

To further evaluate the selected EMA filtering strategy, an additional lightweight temporal filtering baseline, the 1 Euro Filter, was implemented and compared with EMA0.7 using the same win5 reconstructed trajectories. All 26 reconstructed SVO sequences were processed by the compared filters. This additional filtering-baseline comparison was computed from the reprocessed win5 trajectories generated for the revision experiments. Therefore, the values in this subsection are used to compare the temporal filters under the same reprocessed input condition, whereas the earlier EMA ablation summarizes the original module-level temporal filtering experiment. For statistical testing, sequence-level metrics with insufficient valid paired samples or missing values were excluded, and the effective sample size *N* is reported in the statistical comparison table. In the present filtering comparison, the Wilcoxon tests for smoothness, *Z*-axis standard deviation, bone-length standard deviation, and path-length ratio were performed on 23 valid paired sequences.

Table 9 summarizes the overall comparison. Compared with the unfiltered win5 baseline, both EMA0.7 and the 1 Euro Filter reduced the temporal smoothness metric. The 1 Euro Filter produced the strongest smoothing effect, reducing the temporal smoothness metric from 1.321 mm to 0.196 mm. However, its path-length ratio decreased to 0.484, indicating substantial motion attenuation. In contrast, EMA0.7 reduced the temporal smoothness metric to 0.826 mm while maintaining a higher path-length ratio of 0.803. Therefore, EMA0.7 was retained as the final temporal filtering setting because it provided a more balanced trade-off between jitter suppression and motion preservation.

The Wilcoxon signed-rank test further confirmed the filtering effect. EMA0.7 significantly reduced the temporal smoothness metric compared with the win5 baseline. The 1 Euro Filter also significantly reduced the temporal smoothness metric compared with both win5 and EMA0.7. However, the path-length ratio was also significantly lower for the 1 Euro Filter than for EMA0.7, confirming stronger motion attenuation. Table 10 summarizes the paired statistical comparison results.

### 3.5. Evaluation of Bone-Length Consistency Constraint

The third experiment evaluates the effect of the bone-length consistency constraint. Based on the previous experiments, win5+EMA0.7 is used as the input configuration. Two correction strengths are compared: BL0.5 and BL1. The purpose of this experiment is to analyze whether the bone-length constraint can improve skeletal consistency while preserving the reconstructed hand motion.

Table 11 presents the overall quantitative results. In this experiment, win5+EMA0.7 is used as the baseline, and BL0.5 and BL1 are applied on top of this baseline. Compared with the baseline, both BL0.5 and BL1 reduce the bone-length standard deviation. BL0.5 reduces the bone-length standard deviation from 2.0727 mm to 1.1995 mm, while BL1 further reduces it to 0.1915 mm. This indicates that the bone-length constraint effectively improves skeletal structural consistency.

However, stronger correction also introduces larger trajectory modification. Compared with BL0.5, BL1 produces a higher temporal smoothness value, a larger path-length ratio, and a larger correction displacement. Specifically, the correction displacement increases from 0.5571 mm for BL0.5 to 1.8347 mm for BL1. These results suggest that full bone-length projection can over-constrain the reconstructed hand motion, whereas BL0.5 provides a more moderate correction.

**Figure 9:**
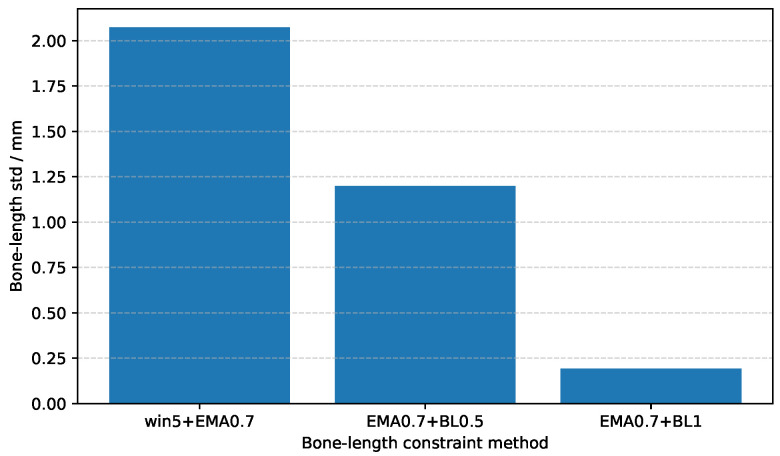
Overall comparison of bone-length standard deviation under different bone-length constraint settings.

Figure 9 compares the overall bone-length standard deviation under different constraint settings. Both BL0.5 and BL1 reduce bone-length variation compared with the baseline, and the reduction is most pronounced for BL1.

**Figure 10:**
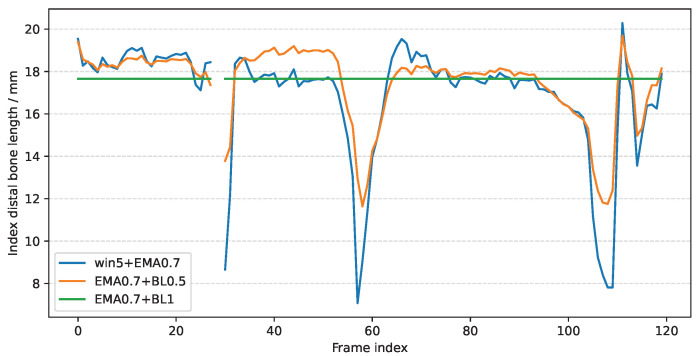
Temporal variation in index distal bone length under different bone-length constraint settings in the B4 sequence.

Figure 10 shows the temporal variation in the distal bone length of the index finger in the B4 sequence. The baseline curve exhibits noticeable fluctuations during dynamic finger motion. BL0.5 suppresses large bone-length deviations while preserving the general temporal trend. In contrast, BL1 almost enforces a constant bone length throughout the sequence, indicating stronger structural correction but reduced flexibility in representing local motion variations.

Table 12 reports the number of best-performing sequences under different bone-length constraint settings. Tied best values are counted for all corresponding methods. BL1 obtains the best bone-length standard deviation in all evaluated sequences, confirming its strong structural correction effect. However, BL0.5 obtains more best-performing sequences in temporal smoothness. The baseline achieves the best path-length ratio and correction displacement because no correction is applied.

Overall, BL1 provides the strongest bone-length consistency but also introduces larger trajectory modification. BL0.5 provides a better trade-off between skeletal consistency and motion preservation. Therefore, BL0.5 is used as the bone-length correction setting in the final pipeline.

### 3.6. Overall Evaluation of the Complete Pipeline

The final experiment evaluates the overall effect of the complete pipeline. Three configurations are compared: win5, win5+EMA0.7, and win5+EMA0.7+BL0.5. The win5 configuration represents the output after local median depth sampling and 3D back-projection. The win5+EMA0.7 configuration further applies temporal filtering, and the win5+EMA0.7+BL0.5 configuration additionally applies the bone-length consistency constraint.

Table 13 summarizes the overall results. Compared with win5, adding EMA 0.7 reduces the temporal smoothness metric from 1.6902 mm to 1.0995 mm, corresponding to a reduction of approximately 34.9%. This indicates that temporal filtering mainly improves trajectory continuity by suppressing frame-to-frame fluctuations. The *Z*-axis standard deviation is also slightly reduced from 8.0328 mm to 7.9664 mm.

After applying BL0.5, the bone-length standard deviation decreases from 2.0727 mm to 1.1995 mm, corresponding to a reduction of approximately 42.1%. This indicates that the bone-length consistency constraint mainly improves skeletal structural stability. The temporal smoothness value changes only slightly from 1.0995 mm to 1.1127 mm, suggesting that moderate bone-length correction does not substantially degrade trajectory smoothness. The *Z*-axis standard deviation increases slightly from 7.9664 mm to 8.1059 mm, indicating that the structural correction primarily affects skeletal consistency rather than depth-direction jitter.

Overall, the three modules contribute to different aspects of the final output. Local median depth sampling provides the landmark-level metric depth for 3D reconstruction, EMA filtering improves temporal continuity, and the bone-length consistency constraint reduces temporal skeletal variation. The final configuration, win5+EMA0.7+BL0.5, provides a balanced setting for stable RGB-D-based 3D hand landmark reconstruction in the evaluated SVO sequences.

### 3.7. Runtime Analysis

Runtime analysis was performed during offline SVO playback on the experimental workstation. The win5 reconstruction stage required 20.45 ms per frame on average, corresponding to approximately 51.20 FPS. The additional computational overhead introduced by the temporal filtering modules was small. EMA0.7 required 0.130 ms per frame, while the 1 Euro Filter required 0.151 ms per frame. Table 14 summarizes the runtime analysis results.

These results indicate that the computational cost of the additional filtering modules is negligible compared with the RGB-D reconstruction stage. However, the current implementation was evaluated in offline SVO playback mode and was not specifically optimized for real-time deployment.

## 4. Discussion

### 4.1. Effect of Local Depth Sampling

The results show that local median depth sampling mainly improves landmark-level depth availability and temporal continuity. Compared with single-pixel sampling, win3 and win5 can use neighboring valid depth values when the center pixel is invalid or affected by local noise. This is particularly useful near fingertips, hand boundaries, and partial self-occlusion regions, where stereo depth maps are more likely to contain holes or foreground–background discontinuities.

However, the advantage of win5 should not be interpreted as universal superiority across all metrics. In the overall comparison, win5 provides the highest valid-depth ratio and the lowest temporal smoothness value, but px still obtains better *Z*-axis standard deviation in some sequences. This suggests that single-pixel sampling can be competitive when the hand surface is locally stable and the landmark falls on a valid depth region. Therefore, the main benefit of win5 is not absolute depth stabilization in every case, but improved robustness to invalid or locally unstable depth measurements.

### 4.2. Trade-Off Between Temporal Smoothing and Motion Preservation

Temporal filtering effectively reduces frame-to-frame fluctuations in the reconstructed 3D landmark trajectories, but stronger smoothing may also attenuate valid hand motion. The comparison among EMA settings showed that smaller EMA parameters produced smoother trajectories but lower path-length ratios. This indicates that the smoothest trajectory is not necessarily the most useful output for hand motion sensing, because excessive smoothing may suppress rapid finger motion, delay response, or distort motion amplitude.

The additional comparison with the 1 Euro Filter further supports this interpretation. The 1 Euro Filter produced the strongest smoothing effect among the compared lightweight filtering methods, but its path-length ratio decreased to 0.484, indicating substantial motion attenuation. In contrast, EMA0.7 provided a less aggressive smoothing effect while maintaining a higher path-length ratio of 0.803. Therefore, EMA0.7 was selected not because it produced the lowest temporal smoothness metric, but because it provided a more practical balance between jitter suppression and motion preservation in the evaluated sequences.

### 4.3. Effect of Bone-Length Consistency Constraint

The bone-length consistency constraint mainly improves the skeletal stability of the reconstructed hand sequence. Since each 3D landmark is independently reconstructed from the sampled depth value, local depth fluctuations may cause the reconstructed bone lengths to vary over time. By estimating a sequence-level reference bone length and correcting child landmarks along the current bone direction, the proposed constraint reduces this temporal skeletal variation.

The comparison between BL0.5 and BL1 further shows the trade-off between structural consistency and motion preservation. BL1 enforces the strongest bone-length consistency and achieves the lowest bone-length standard deviation. However, it also introduces a larger correction displacement and a higher path-length ratio, indicating that the reconstructed trajectory is more strongly modified. In contrast, BL0.5 reduces the bone-length standard deviation from 2.0727 mm to 1.1995 mm while keeping the correction displacement relatively small. Therefore, BL0.5 is more suitable as a moderate structural correction setting for the final RGB-D sensing front-end.

It should be emphasized that the reduced bone-length variation should be interpreted as improved temporal skeletal consistency rather than improved absolute 3D landmark accuracy. Since the bone-length constraint explicitly modifies child landmark positions along the current bone direction, it may also alter the reconstructed motion. Therefore, the correction strength should be selected as a trade-off between structural consistency and motion preservation.

### 4.4. Difference from Learning-Based Hand Pose Estimation

The proposed method is not intended to replace learning-based 3D hand pose estimation models, including recent RGB-D fusion and topology-aware hand pose estimation methods [14,19]. Learning-based methods based on voxel representations, point clouds, compact pose priors, or parametric hand models are usually designed to predict complete 3D hand pose from RGB, depth, or RGB-D inputs. These methods are typically evaluated on benchmark datasets with ground-truth 3D annotations.

In contrast, this work focuses on a lightweight RGB-D sensing front-end that combines an existing semantic landmark detector with metric stereo depth measurements. The objective is to obtain stable metric 3D landmark trajectories through geometric back-projection and lightweight temporal and structural regularization, without training a new hand pose regression network. Therefore, the evaluation emphasizes depth availability, temporal stability, skeletal consistency, and motion preservation rather than benchmark-level pose accuracy. This positioning is consistent with the intended use of the proposed method as a practical front-end for low-cost hand motion measurement and interaction prototyping.

### 4.5. Practical Implications for Low-Cost RGB-D Hand Motion Sensing

The proposed pipeline provides a practical way to combine semantic RGB-based landmarks with metric stereo depth. MediaPipe contributes stable landmark semantics and a fixed hand topology, while the ZED2 camera provides metric depth for camera-frame 3D reconstruction. The local median sampling strategy improves the robustness of landmark-level depth acquisition, EMA filtering improves temporal continuity, and the bone-length constraint improves skeletal consistency.

Another practical advantage is that the method does not require additional model training or expensive marker-based motion capture equipment. The complete pipeline can be implemented using a commercial RGB-D stereo camera and open-source vision tools. This makes it suitable for low-cost hand motion measurement, human–computer interaction experiments, robot teleoperation front-end development, and preliminary hand motion analysis. Nevertheless, the current results should be interpreted as system-level stability validation rather than evidence of absolute 3D pose accuracy.

### 4.6. Limitations

Several limitations should be noted. First, the current hand-motion experiments were conducted on a self-collected dataset recorded from a single subject under controlled indoor conditions. This design was useful for controlled module-level ablation, but it does not fully evaluate generalization across different users, hand sizes, skin tones, lighting conditions, backgrounds, viewpoints, and camera-to-hand distances. Therefore, the current hand-motion results should be interpreted as preliminary system-level validation rather than large-scale benchmark evaluation.

Second, although the revised manuscript includes a checkerboard-based calibrated reference-distance validation experiment, this validation evaluates the metric depth sampling and 3D back-projection component using a rigid planar target. It does not provide motion-capture-level ground truth for articulated hand landmarks. Therefore, the reported hand-motion results mainly support improvements in depth availability, temporal stability, skeletal consistency, and motion preservation, rather than absolute 3D hand landmark accuracy. Future work should introduce motion-capture systems, calibrated wearable markers, or other external reference measurements to evaluate the absolute 3D error of articulated hand landmarks.

Third, severe occlusion and hand–object interaction remain challenging. When MediaPipe fails to detect landmarks or the ZED2 depth map contains large invalid regions, the proposed local depth sampling and post-processing modules cannot fully recover missing 3D information. In addition, the current bone-length constraint only enforces a simple skeletal consistency prior and does not model full hand kinematics, joint-angle limits, or object contact. Future work will include multi-subject validation, real-time deployment evaluation, stronger kinematic constraints, and more challenging manipulation scenarios.

## 5. Conclusions

This paper presented a low-cost RGB-D sensing front-end for stable RGB-D-based hand landmark reconstruction by combining MediaPipe semantic hand landmarks with ZED2 stereo depth. The proposed pipeline obtains landmark-level metric depth from the aligned stereo depth map, reconstructs 3D landmarks through camera back-projection, and further applies EMA-based temporal filtering and a bone-length consistency constraint to improve the temporal and structural stability of the reconstructed hand landmark sequence.

Experiments on a self-collected SVO dataset showed that the proposed modules contribute to different aspects of the final output. The 5×5 local median sampling strategy slightly improved depth availability and temporal continuity compared with single-pixel sampling, with statistical testing showing a significant reduction in the temporal smoothness metric. EMA 0.7 provided a practical trade-off between jitter suppression and motion preservation. The BL0.5 bone-length constraint reduced bone-length variation while introducing limited trajectory modification. The final configuration, win5+EMA0.7+BL0.5, provided a balanced setting for the evaluated RGB-D hand landmark sequences.

The current results should be interpreted as system-level evidence of improved temporal stability and skeletal consistency under controlled indoor conditions, rather than as full validation of absolute 3D hand landmark accuracy. Future work will include multi-subject validation, more diverse recording conditions, real-time deployment evaluation, and external ground-truth measurements such as calibrated markers or motion-capture systems to assess absolute 3D reconstruction error. In addition, stronger kinematic constraints and hand–object interaction modeling will be explored for more challenging manipulation scenarios.

## Figures and Tables

**Table 1:** Experimental actions and scenarios in the self-collected SVO dataset.

Category	ID	Action Name	Scenario Description	Duration (s)	Reps	Purpose
**Static**	A1	static_open_center	Open hand at the frame center	10	2	Baseline stability
	A2	static_pinch_center	Thumb–index pinch posture	10	2	Fingertip posture
	A3	static_half_fist_center	Half-fist posture	10	2	Finger bending
	A4	static_open_edge	Open hand near the image edge	10	2	Edge-view stability
**Dynamic**	B1	move_left_right	Left–right hand translation	10	2	Translation
	B2	move_near_far	Near–far depth movement	10	2	Depth variation
	B3	rotate_wrist	Front-to-side wrist rotation	10	2	Viewpoint change
	B4	open_close_fingers	Repeated finger opening and closing	10	2	Finger motion
**Occlusion**	C1	self_occlusion_side	Side rotation with self-occlusion	10	2	Self-occlusion
	C2	partial_occlusion_index	Partial index-finger occlusion	10	2	Partial occlusion
	C3	grasp_simple_object	Simple object grasping	10	2	Object interaction
**Recovery**	D1	fast_motion	Rapid hand or finger motion	8	2	Fast motion
	D2	leave_and_return	Hand leaving and re-entering frame	8	2	Re-detection

**Table 2:** Evaluation metrics used in the experiments.

Metric	Field	Description	Preference
Detection rate	detected_rate	Ratio of frames with successful MediaPipe hand detection	Higher
Valid-depth ratio	mean_valid_depth_ratio	Ratio of detected landmarks with valid ZED2 depth values	Higher
*Z*-axis std.	mean_std_Z	Depth-direction jitter of reconstructed 3D landmarks	Lower
Temporal smoothness	mean_smooth_xyz	Mean second-order difference of 3D landmark trajectories	Lower
Bone-length std.	mean_bone_std	Temporal variation in reconstructed hand bone lengths	Lower
Path-length ratio	path_length_ratio	Ratio of processed trajectory length to baseline trajectory length	Closer to 1
Correction displacement	mean_correction	Mean landmark displacement introduced by bone-length correction	Lower

**Table 3:** Overall quantitative results of different depth sampling strategies.

Method	DetectionRate	Valid-DepthRatio	*Z*-Std. (mm)	Smoothness(mm)	Bone Std.(mm)
px	0.8058	0.9731	**8.0307**	1.7163	**2.2263**
win3	0.8058	0.9736	8.0327	1.7023	2.2366
win5	0.8058	**0.9738**	8.0328	**1.6902**	2.2388

Note: Higher values are preferred for detection rate and valid-depth ratio, whereas lower values are preferred for *Z*-std., smoothness, and bone std. Bold values indicate the best-performing result for each metric.

**Table 4:** Number of best-performing sequences under different evaluation metrics.

Metric	Criterion	N	px	win3	win5
Valid-depth ratio	Higher	11	9	10	11
*Z*-axis std.	Lower	11	5	3	3
Temporal smoothness	Lower	11	0	1	10
Bone-length std.	Lower	11	3	2	6

**Table 5:** Paired Wilcoxon signed-rank test results for the 5×5 local median depth sampling strategy compared with single-pixel sampling.

Metric	*N* Valid Pairs	Mean Difference	*p*-Value
Valid-depth ratio	11	+0.000759	0.500000
*Z*-axis std.	11	+0.002159 mm	0.577148
Smoothness	11	−0.026197 mm	0.001953
Bone std.	11	+0.012485 mm	0.413086

Note: *N* denotes the number of valid paired segments used in the paired Wilcoxon signed-rank test. Two segments with missing sequence-level metric values were excluded from the statistical comparison.

**Table 6:** Calibrated reference-distance validation results using the checkerboard board.

Metric	Value
Number of SVO sequences	5
Number of valid distance measurements	542,276
Mean valid corner ratio	1.000
MAE	0.181 mm
RMSE	0.225 mm
Mean signed error	−0.150 mm
Error std.	0.168 mm
Median absolute error	0.156 mm
Maximum absolute error	1.099 mm

**Table 7:** Overall quantitative results of temporal filtering.

Method	*Z*-Std.	Smoothness	Bone Std.	Path Ratio
	(mm)	(mm)	(mm)	
win5	8.0328	1.6902	2.2388	1.0000
EMA 0.3	7.7773	0.4950	2.2934	0.5622
EMA 0.5	7.9034	0.7802	2.1841	0.6819
EMA 0.7	7.9664	1.0995	2.1681	0.7999

**Table 8:** Number of best-performing sequences under different temporal filtering metrics.

Metric	Criterion	N	win5	EMA 0.3	EMA 0.5	EMA 0.7
*Z*-axis std.	Lower	11	5	6	0	0
Temporal smoothness	Lower	11	0	11	0	0
Bone-length std.	Lower	11	1	7	1	2
Path-length ratio	Closer to 1	13	13	0	0	0

**Table 9:** Comparison between EMA0.7 and the 1 Euro Filter using the win5 reconstructed trajectories.

Method	*Z*-Std.	Smoothness	Bone Std.	Path Ratio	Overhead
	(mm)	(mm)	(mm)		(ms/Frame)
win5 raw	7.399	1.321	1.790	1.000	0.000
EMA0.7	7.313	0.826	1.693	0.803	0.130
1 Euro Filter	7.006	0.196	1.678	0.484	0.151

**Table 10:** Paired Wilcoxon signed-rank test results for temporal filtering comparison.

Comparison	Metric	*N*	*p*-Value
EMA0.7 vs. win5 raw	Smoothness	23	$2.38\times 10^{-7}$
EMA0.7 vs. win5 raw	Path ratio	23	$2.38\times 10^{-7}$
1 Euro vs. win5 raw	Smoothness	23	$2.38\times 10^{-7}$
1 Euro vs. win5 raw	Path ratio	23	$2.38\times 10^{-7}$
1 Euro vs. EMA0.7	Smoothness	23	$2.38\times 10^{-7}$
1 Euro vs. EMA0.7	Path ratio	23	$2.38\times 10^{-7}$

**Table 11:** Overall quantitative results of the bone-length consistency constraint.

Method	*Z*-Std.	Smoothness	Bone Std.	Path Ratio	Correction
	(mm)	(mm)	(mm)		(mm)
Baseline	7.9664	1.0995	2.0727	1.0000	0.0000
+BL0.5	8.1059	1.1127	1.1995	1.0157	0.5571
+BL1	8.6542	1.9717	0.1915	1.2682	1.8347

**Table 12:** Number of best-performing sequences under different bone-length constraint settings.

Metric	Criterion	N	win5+EMA0.7	BL0.5	BL1
*Z*-axis std.	Lower	11	3	3	5
Temporal smoothness	Lower	11	3	7	1
Bone-length std.	Lower	11	0	0	11
Path-length ratio	Closer to 1	13	13	0	0
Correction displacement	Lower	13	13	0	0

**Table 13:** Overall comparison of the complete RGB-D-based 3D hand landmark reconstruction pipeline.

Method	*Z*-Std.	Smoothness	Bone Std.
	(mm)	(mm)	(mm)
win5	8.0328	1.6902	2.2388
win5+EMA0.7	7.9664	1.0995	2.0727
win5+EMA0.7+BL0.5	8.1059	1.1127	1.1995

**Table 14:** Runtime analysis during offline SVO playback for the RGB-D reconstruction and temporal filtering modules.

Module	Processing Time	Estimated FPS/Overhead
win5 reconstruction	20.45 ms/frame	51.20 FPS
EMA0.7 filtering	0.130 ms/frame	Additional overhead
1 Euro Filter	0.151 ms/frame	Additional overhead

## Data Availability

The processed data supporting the findings of this study are available from the corresponding author upon reasonable request. The raw SVO files are not publicly available due to their large file size.

## Abbreviations

The following abbreviations are used in this manuscript:

RGB-D	Red–green–blue-depth
SVO	Stereo video object
EMA	Exponential moving average
BL	Bone-length constraint
HCI	Human–computer interaction
